# 298 Genomic Epidemiology Demonstrates that Decolonization reduces Staphylococcus aureus transmission in a Neonatal Intensive Care Unit

**DOI:** 10.1017/ash.2026.10451

**Published:** 2026-06-23

**Authors:** Kayla Ruch, Mayar Al Mohajer

**Affiliations:** 1 Becton Dickinson (BD); 2 Baylor College of Medicine

## Abstract

**Background:** Infection preventionists (IPs) increasingly lead systemwide healthcare epidemiology functions integrating infection prevention, antimicrobial stewardship, and preparedness for emerging pathogens. The Advanced Leadership Certification in Infection Prevention and Control (AL-CIP) is the first credential to formally assess advanced IPC leadership competencies, yet the leadership practices, epidemiologic decision-making, and risk management strategies employed by certified professionals have not been empirically described. **Methods:** The Leadership Evaluation and Development for Infection Preventionists (LEAD-IP) study used an observational nested cohort design. Secondary portfolio data from AL-CIP applicants across two 2025 certification cycles were analyzed to characterize demographic and professional attributes. A nested cohort of certified professionals (18%) completed a structured 60-minute qualitative interview via Microsoft Teams. Interviews followed the IRB-approved 34-item LEAD-IP instrument assessing leadership behaviors, healthcare epidemiology decision-making, antimicrobial stewardship engagement, risk assessment practices, communication strategies, and preparedness for emerging pathogens. Data were managed in REDCap and analyzed using Braun and Clarke’s inductive thematic analysis with a structured codebook, triangulation, and verification of thematic saturation. **Results:** Thirty AL-CIP–certified leaders across healthcare systems, public health agencies, and academic settings participated. Across seven themes, participants described a shift from task-based practice to systems-level healthcare epidemiology leadership, integrating surveillance data, antimicrobial resistance patterns, and organizational risk indicators into strategic direction-setting. Leaders emphasized dashboards, analytic tools, and improvement models to detect outbreaks earlier, evaluate stewardship interventions, and guide resource allocation. Transparent communication and psychological safety facilitated early escalation of HAI clusters, antibiotic misuse, environmental risks, and emerging pathogen concerns such as Candida auris. Participants reported persistent public health infrastructure limitations, including fragmented reporting pathways, inconsistent data-sharing with health departments, and delays in organism identification that impeded timely epidemiologic response. Significant antibiotic stewardship gaps were also identified—particularly limited analytic capacity, insufficient infectious disease support, and low organizational prioritization. Structural barriers such as staffing shortages and limited protected time constrained proactive IPC and stewardship leadership. Inclusive engagement of under-resourced teams improved situational awareness and intervention success. Proactive risk management—monitoring construction, airflow disruptions, MDRO trends, and public health alerts—was viewed as essential. AL-CIP certification strengthened leadership identity, credibility, and strategic influence. **Conclusions:** AL-CIP–certified professionals demonstrated advanced capabilities in healthcare epidemiology, antibiotic stewardship, and public health preparedness through systems thinking, data-driven decision-making, inclusive engagement, and proactive risk management. The AL-CIP credential enhanced leaders’ confidence and organizational impact, supporting a more resilient and prepared healthcare epidemiology workforce.

